# Strong Dispersal Limitation of Microbial Communities at Shackleton Glacier, Antarctica

**DOI:** 10.1128/msystems.01254-22

**Published:** 2023-01-31

**Authors:** Nathan P. Lemoine, Byron J. Adams, Melisa Diaz, Nicholas B. Dragone, André L. C. Franco, Noah Fierer, W. Berry Lyons, Ian D. Hogg, Diana H. Wall

**Affiliations:** a Department of Biological Sciences, Marquette University, Milwaukee, Wisconsin, USA; b Department of Zoology, Milwaukee Public Museum, Milwaukee, Wisconsin, USA; c Department of Biology, Evolutionary Ecology Laboratories, Monte L. Bean Museum, Brigham Young University, Provo, Utah, USA; d School of Earth Sciences, Byrd Polar and Climate Research Center, The Ohio State University, Columbus, Ohio, USA; e Department of Ecology and Evolutionary Biology, Cooperative Institute for Research in Environmental Sciences, University of Colorado at Boulder, Boulder, Colorado, USA; f Department of Biology, Colorado State University, Fort Collins, Colorado, USA; g Canadian High Arctic Research Station, Polar Knowledge Canada, Cambridge Bay, Nunavut, Canada; h School of Science, University of Waikato, Hamilton, New Zealand; i School of Global Environmental Sustainability, Colorado State University, Fort Collins, Colorado, USA; Swansea University

**Keywords:** community assembly, stochasticity, determinism, niche, dispersal

## Abstract

Microbial communities can be structured by both deterministic and stochastic processes, but the relative importance of these processes remains unknown. The ambiguity partly arises from an inability to disentangle soil microbial processes from confounding factors, such as aboveground plant communities or anthropogenic disturbance. In this study, we characterized the relative contributions of determinism and stochasticity to assembly processes of soil bacterial communities across a large environmental gradient of undisturbed Antarctic soils. We hypothesized that harsh soils would impose a strong environmental selection on microbial communities, whereas communities in benign soils would be structured largely by dispersal. Contrary to our expectations, dispersal was the dominant assembly mechanism across the entire soil environmental gradient, including benign environments. The microbial community composition reflects slowly changing soil conditions and dispersal limitation of isolated sites. Thus, stochastic processes, as opposed to deterministic, are primary drivers of soil ecosystem assembly across space at our study site. This is especially surprising given the strong environmental constraints on soil microorganisms in one of the harshest environments on the planet, suggesting that dispersal could be a driving force in microbial community assembly in soils worldwide.

**IMPORTANCE** Because of their diversity and ubiquity, microbes provide an excellent means to tease apart how natural communities are structured. In general, ecologists believe that stochastic assembly processes, like random drift and dispersal, should dominate in benign environments while deterministic processes, like environmental filtering, should be prevalent in harsh environments. To help resolve this debate, we analyzed microbial community composition in pristine Antarctic soils devoid of human influence or plant communities for eons. Our results demonstrate that dispersal limitation is a surprisingly potent force of community limitation throughout all soil conditions. Thus, dispersal appears to be a driving force of microbial community assembly, even in the harshest of conditions.

## INTRODUCTION

Ever since Darwin published *On the Origin of Species* (1), ecologists have sought to understand the processes that control community composition. In general, communities can be assembled via either deterministic or stochastic processes (2, 3). Deterministic processes arise from niche theory, including both competitive exclusion (i.e., selection against overly similar species from occupying a community [4]) and environmental filtering (i.e., selection against overly dissimilar species when the environment is unsuitable [5]). Stochastic assembly, on the other hand, refers to probabilistic processes like dispersal, priority effects during colonization, and community drift via random births and deaths (2). There is as yet no clear trend as to whether determinism or stochasticity are more important to community structure. The relative importance of determinism and stochasticity varies among both studies and locations (3, 6–13). The balance between determinism and stochasticity is likely dictated by the abiotic environment but few studies explicitly link changes in community processes to changes in the environment using gradient studies (8, 10, 14). Identifying how community assembly mechanisms vary spatially has thus been described as one of the most important goals in ecology.

The ways in which community assembly mechanisms vary along environmental gradients remains contested due to a number of contrasting theories and results. On one hand, some have suggested that harsh environments should be stochastic and benign environments should be structured by deterministic competition (12). Indeed, competition among plant species is strongest in warm, resource-rich environments compared to cold and stressful environments (15). On the other hand, studies suggest that deterministic processes, such as environmental filtering, should also be strongest in harsh environments that impose severe constraints on occupancy and growth (16). For example, environmental filtering of plant communities was strongest in old-growth forests where light and nutrients were limiting (17). It is also possible that determinism is strongest in both harsh and resource-rich environments, while stochasticity dominates in the middle of the resource gradient, as implied by the stress-gradient hypothesis (18). Only a quantitative gradient study that allows determinism and stochasticity to vary in strength can test these hypotheses.

Soil microbial communities provide an excellent system to test assembly theory because microbial communities are incredibly speciose, omnipresent even in the harshest environments, and exhibit high spatial and temporal turnover (19). Microbes were historically thought to be uninhibited by dispersal limitation, as stated in the adage “everything is everywhere but the environment selects” (20). However, the Baas-Becking hypothesis has now been superseded by shifting emphasis toward resolving the “neutral versus niche” debate (21), including how stochastic and deterministic processes vary in strength with environmental conditions. Unfortunately, efforts to differentiate between determinism and stochasticity are complicated by two issues. First, a single mechanism can yield both deterministic and stochastic patterns. For example, dispersal constraints are typically considered stochastic, but can yield beta-diversity patterns that appear deterministic (5). Likewise, competition is generally thought to result in deterministic patterns of similarity; however, competition can also result in stochastic assembly even when niche processes are the driving force (22). Second, many null modeling methods partition total amounts of variation in beta-diversity into deterministic and stochastic components. Doing so forces determinism and stochasticity to trade off because an increase in the variability explained by one factor must be matched by a decrease in variation explained by the other. Yet there is no a *priori* reason to assume a zero-sum balance between determinism and stochasticity.

**FIG 1:**
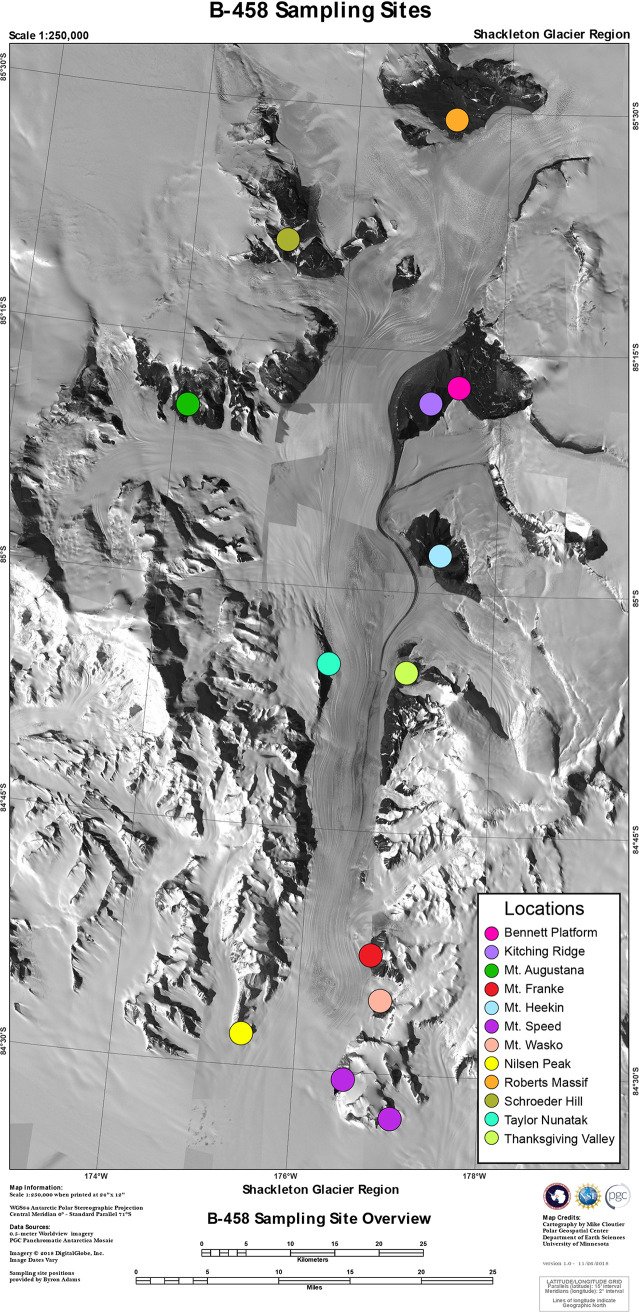
Map of the Shackleton Glacier, with sampling sites denoted as colored point. Map by Mike Cloutier, Polar Geospatial Center (Imagery © 2021 Maxar; reproduced with permission).

Here, we tested how community assembly mechanisms varied along an environmental gradient of soil conditions in the Shackleton Glacier region of Antarctica (Fig. 1). Specifically, we tested three hypotheses. Hypothesis 1: Both the environment and spatial patterns influence microbial community composition. This would indicate that community assembly is determined by a balance of both putative deterministic (i.e., environmental filtering) and stochastic (i.e., dispersal) mechanisms. Hypothesis 2: Deterministic assembly mechanisms are strongest in the harshest soils, but become less important as soil stress declines. Hypothesis 3: Tradeoffs between environmental filtering and dispersal are directly responsible for the observed changes in assembly mechanisms. We tested Hypotheses 2 and 3 by combining the power of traditional null modeling methods with a new model that explicitly accounts for different mechanisms and does not implicitly assume a trade-off between factors. By working with Antarctic soils, our study presents a novel ecosystem found nowhere else on the planet. First, the stability of Antarctic soils is unique compared to almost every other location on Earth (23), which have undergone extreme changes within the last 2 million years. Second, our study area has been devoid of aboveground plants since the Neogene (2.5 million years before present) and possibly the mid-Miocene (14 million years before present) (24, 25), such that we can test how changes in soil properties per se influence microbial assembly patterns. Finally, we used a new modeling approach to explicitly identify the relative importance of dispersal versus environment, providing a detailed assessment of assembly mechanisms. Our approach also expands on previous work by quantifying independent assembly mechanisms for colonization (i.e., presence/absence) and postcolonization success (i.e., relative abundance).

## RESULTS

### Hypothesis 1: The environment and spatial arrangement control microbial community composition.

**FIG 2:**
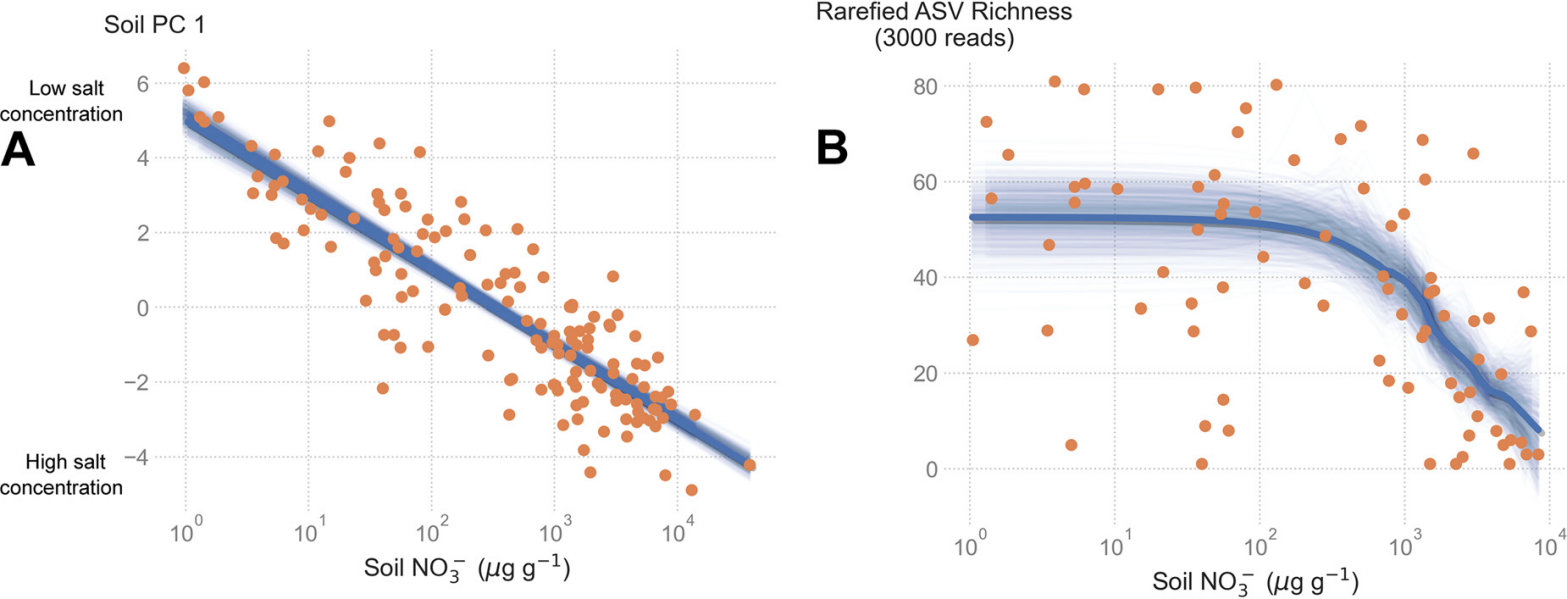
Relationships between soil salt concentrations, exposure age, and microbial ASV richness. (A) The relationship between soil principal component (PC 1) and soil NO_3_- concentrations. The thick blue line shows the linear model fit to the observed data, and thin blue lines show 1,000 bootstrapped linear regressions. (B) Relationship between ASV richness and soil NO_3_- concentrations. Thick blue line shows the LOWESS fit to the observed data, while thin blue lines show 1,000 bootstrapped LOWESS estimates.

Both the environment and spatial distribution of sites controlled microbial community composition. Microbial species richness in particular was strongly regulated by the environment, but only in the harshest soils. Ordination of soil geochemistry yielded a single axis (Soil PC 1) that explained 56% of variation in geochemical and physical properties (Table S1). Soil PC 2 explained an additional 12% of geochemical variation among sites. Concentrations of Ca^2+^, Cl-, F-, K^+^, Mg^2+^, Na^+^, NH_3_, NO_3_-, PO_4_^3−^, SiO_2_, SO_4_^2−^, and Sr^2+^ all positively co-varied, clustering along Soil PC 1. Thus, Soil PC 1 was a salt concentration gradient that could be adequately represented by soil NO_3_- concentrations (pseudo-R^2^ = 0.82, Fig. 2A). As a result, we hereafter refer to soil NO_3_- concentrations to represent the soil salt gradient.

10.1128/msystems.01254-22.6 TABLE S1 Variable loadings (distance calculation) of each variable on the first two principal component axes. Download Table S1, DOCX file, 0.01 MB. Copyright © 2023 Lemoine et al. 2023 Lemoine et al. https://creativecommons.org/licenses/by/4.0/ This content is distributed under the terms of the Creative Commons Attribution 4.0 International license.

The environment imposed a strong constraint on microbial community composition, as evidenced by the effect of soil NO_3_- on microbial diversity. Rarefied richness of amplicon sequence variants (R-ASV richness) appeared to be determined in large part by soil NO_3_- concentrations (i.e., the environment, Fig. 2B). Harsh soils with high NO_3_- concentrations had fewer ASVs present than benign environments (pseudo-R^2^ = 0.33, Fig. 2B). Mean R-ASV richness was stable (~53 ASVs) for soils with low NO_3_- concentrations until a threshold around 10^3^* μg* g^−1^ NO_3_-, beyond which R-ASV decline steeply (pseudo-R^2^ = 0.40, Fig. 2B).

**FIG 3:**
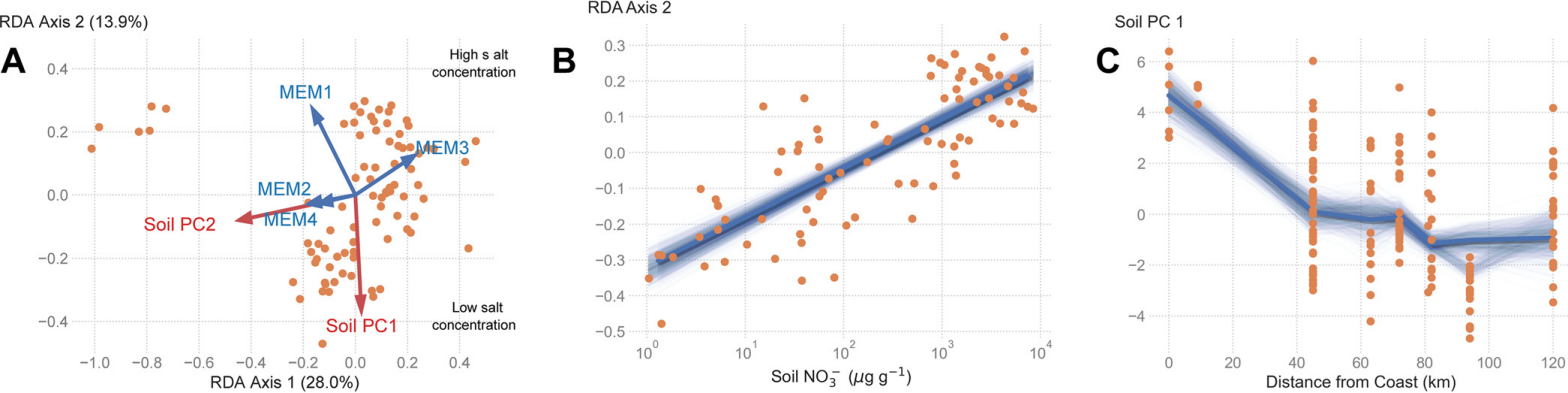
Redundancy analyses of microbial community composition. (A) Redundancy analysis (RDA) biplot of microbial community composition in relation to physical variables and spatial arrangement of sites. The cluster of points in the upper left corner are Robert’s Massif. (B) Relationship between RDA Axis 2 and soil NO_3_- concentrations. Thick blue line shows the linear model fit to all observed data, and thin blue lines show 1,000 bootstrapped linear regressions. (C) Relationship between Soil PC 1 and distance from the coast (km) for each site. Thick blue line shows the LOWESS fit to all observed data, and thin blue lines show 1,000 bootstrapped LOWESS fits.

Redundancy analysis (RDA) demonstrated that microbial community composition was also constrained by both the environment and spatial site arrangement. RDA 1 represented both dispersal and environmental effects (Fig. 3A). In particular, RDA 1 separated the location closest to the polar plateau (Robert’s Massif) from all other sites (based on MEM3), whereas RDA 2 reflected the salt concentration gradient and characterized sites based on their distance from the coast (based on MEM1). Spatially, this means that inland sites further from the cost, particularly those on Robert’s Massif, had distinct microbial communities from low-elevation coastal sites. Environmentally, given that soil NO_3_- concentrations are a good proxy for soil saltiness (Fig. 2A), RDA 2 illustrated the degree to which soil salt concentrations influence microbial community composition (Fig. 3B). The similarity in soil salt concentrations among sites close to and distant from the coast suggests that the microbial uniqueness of the polar plateau sites stemmed from spatial isolation rather than changes in soil chemistry (Fig. 3C). Variance partitioning confirmed that both location and soil characteristics structure microbial communities. Environmental variables accounted for 16% of the variation in soil microbial communities, while spatial variables accounted for an additional 16% of variability. The covariation of soil characteristics along the spatial gradient explained another 9% of community variability.

### Hypothesis 2: Deterministic processes should be strongest in the harshest soils.

**FIG 4:**
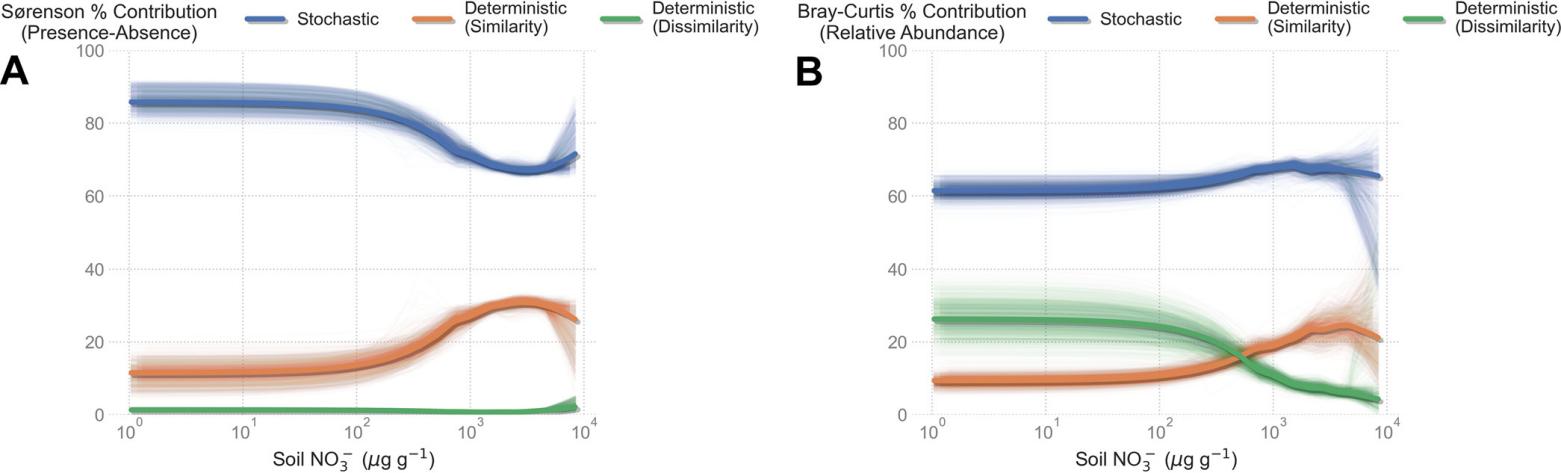
Microbial communities were largely structured by stochastic processes across all soil wetting ages. (A) Contribution of stochastic and deterministic processes to microbial community composition based on Sørenson dissimilarity, which records presence-absence dissimilarities. We calculated the average variance component for each site. Thick lines show a LOWESS regression against NO_3_- concentrations. Thin lines show 1,000 bootstrapped LOWESS regressions. (B) Contribution of stochastic and deterministic processes to microbial community composition based on Bray-Curtis dissimilarities, which record relative abundance dissimilarities. In all simulations, phylum richness was held fixed at the observed level for each site. Thick lines show a LOWESS regression against NO_3_- concentrations. Thin lines show 1,000 bootstrapped LOWESS regressions.

We tested the hypothesis that deterministic processes, like environmental filtering, should be strongest in the harshest soils using a null-modeling approach. We found that the relative contributions of stochastic and deterministic processes to community assembly changed across the soil salt concentration gradient, but not in the predicted way. In less stressful environments, stochastic processes contributed to the bulk of presence-absence composition (~90%), while deterministic similarity (i.e., environmental filtering) contributed the remaining variability (Fig. 4A). The relative importance of stochastic and deterministic assembly remained constant across the salt gradient up to 10^3^ μg g^−1^ NO_3_- (Fig. 4A). In saltier soils, the contribution of stochastic processes declined, as expected, and also became more variable. However, stochastic processes still contributed 60% to 70% of the presence/absence variability. Deterministic environmental filtering, while important, was never the dominant process (Fig. 4). Results for relative taxon abundances were less certain due to the added complexities of predicting species abundance, but they exhibited patterns similar to the phylum-level analyses presented here (see supplemental information). In the most benign soils, stochastic processes contributed ~60% of the variation in microbial community turnover, while deterministic dissimilarity and similarity each averaged about 30% and 10% of the contribution, respectively (Fig. 4B). In the saltiest soils, the contribution of stochasticity increased to ~65%, while the importance of deterministic dissimilarity declined to negligible levels and was replaced entirely by deterministic similarity (Fig. 4B). These results suggest that less harsh environments are potentially regulated more via niche partitioning while stressful soils become increasingly structured via environmental filtering, but stochastic processes were the dominant structuring force across the entire soil gradient.

### Hypothesis 3: Environmental filtering should be stronger than dispersal in the harshest soils.

**FIG 5:**
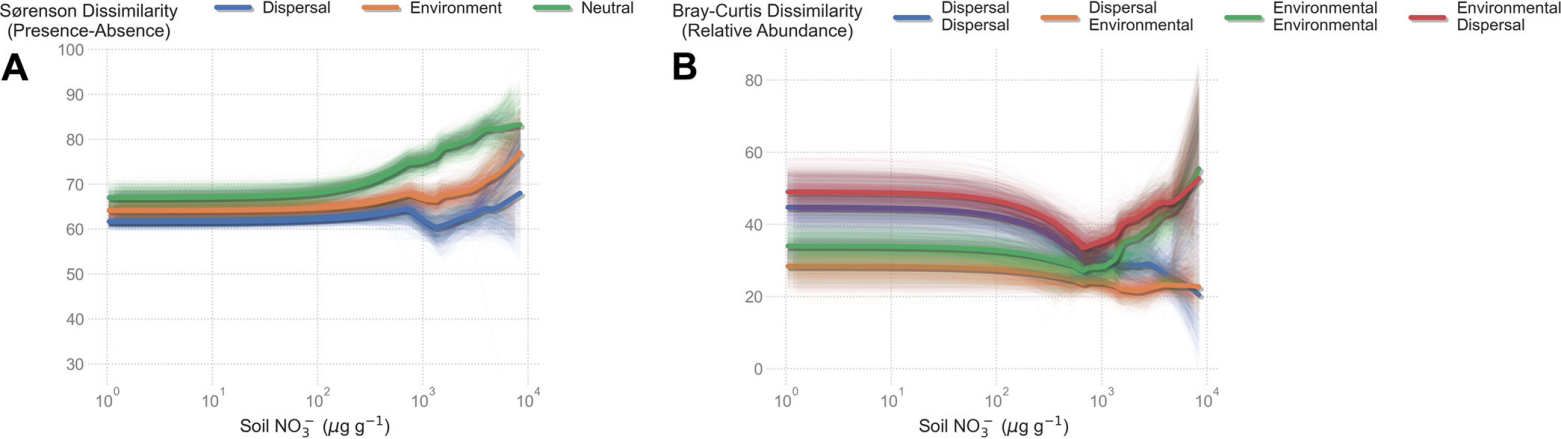
Dissimilarity of modeled communities to observed communities when modeled communities were structured by neutral, dispersal, or environmental processes. (A) Averaged Sørenson dissimilarity of simulated communities from observed communities. For each site, we took the average pairwise dissimilarities to the observed site from 1,000 model simulations. Thick lines show the LOWESS fit to the averaged dissimilarities as a function of soil NO_3_- concentrations. (B) For each site, we took the average pairwise dissimilarities to the observed site from 1,000 model simulations. Thick lines show the LOWESS fit to the average dissimilarities as a function of soil NO_3_- concentrations. Thin lines show 1,000 bootstrap simulations. In all simulations, phylum richness was fixed to the observed level for each site.

We expected to find strong environmental filtering and weak dispersal limitation in most stressful soils with extremely high soil salt concentrations. However, our models did not support this hypothesis. With presence/absence data, the importance of dispersal increased with soil NO_3_- concentrations (Fig. 5A). Below 10^3^ μg g^−1^ NO_3_-, neutral, environmental, and dispersal weightings all resulted in simulated communities that were 60% to 70% dissimilar from observed communities (Fig. 5A). Above 10^3^ μg g^−1^ NO_3_-, neutral weighting and environmental weighting provided poorer fits to the data, through dispersal weighting maintained a 60% to 70% dissimilarity across all relative exposure ranges (Fig. 5A). This confirms that both environmental filtering and dispersal increase in importance as soils develop harsher environmental conditions, but dispersal remains the dominant factor for the presence/absence of microbial taxa in Antarctic soils.

Simulations that included relative abundance based on Bray-Curtis dissimilarities yielded a slightly different interpretation. First, Bray-Curtis simulations fit the observed data much better than presence/absence data, with dissimilarities ranging from 20% to 60% (Fig. 5B). In other words, it mattered less whether taxa occurrence was dictated by dispersal or the environment, so long as abundance was determined by the environment. Above the 10^3^ μg g^−1^ NO_3_- threshold, patterns reversed. The weightings for presence/absence became the most important factor, as both dispersal-weighted colonization models were ~40% more accurate than models with environmentally weighted colonization, regardless of abundance (Fig. 5B). Despite changes in the goodness of fit for some models, the best-fitting model remained consistent across the entire salt concentration gradient; dispersal-limited presence/absence and environmental-limited abundance consistently provided the best fits to observed community composition (Fig. 5B). From these results, we can conclude that, regardless of soil salt concentrations, dispersal is the primary factor determining which species appear in a community, while the environment determines how well those species perform once present.

## DISCUSSION

Our data highlight the importance of extreme environmental gradients, as changes in soil microbial diversity only manifested in extremely harsh environments. Previous studies of community assembly demonstrated that deterministic and stochastic processes trade off, with stochastic processes dominating in benign soils and determinism increasing in harsher, low-nutrient soils (8, 10, 14). In Antarctic soils, salt concentrations form the basis of a stress gradient (Fig. 2), and thus, we expected environmental filtering to be the predominant force structuring microbial communities. Using diversity-based modeling approaches, we did indeed find evidence for strengthened deterministic processes in stressful soils, coupled with weakening stochastic effects (Fig. 4). Yet our modeling approach detected a consistently strong signal of dispersal limitation in soils salt concentration (Fig. 5). These lines of evidence are not contradictory, as stochastic processes consistently explained ~60% of the variation in community assembly, even in stressful soils, and the trade-off in determinism could in fact be due to a decline in other effects, such as drift (26).

Our modeling approach makes two key assumptions: that dispersal limitation can accurately be represented by geographic ranges, and that we measured all relevant environmental parameters. Regarding the assumption that bacterial dispersal is correlated with geographic distance, the degree of dispersal in bacterial communities is still an open question. Some studies have documented near-global dispersal of certain microbial taxa in the atmosphere (27), leading some researchers to propose that microbes are never limited by geographic barriers (28). Yet numerous experimental studies have documented dispersal limitation over very short scales (<1 km) for fungi (29, 30), bacteria (31), and archaea (32). The assumption that dispersal limitation could be represented as geographic separation therefore is reasonable. Our models imply that dispersal is an important process, because our models that included geographic dispersal limitation consistently outperformed other models. The second assumption underlying our support vector machine approach to model environmental niches is that we have measured all appropriate parameters. Our environmental parameters include soil water and salt concentrations, all of which are known to be strong determinants of Antarctic soil community composition (33–35). We are, therefore, confident that our support vector machine model can appropriately quantify relative habitat suitability for each taxon.

Our study represents a comprehensive examination of microbial community assembly, free from confounding factors due to the uniqueness of Antarctic soils remaining stable and free from anthropogenic disturbance for up to several million years. However, this stability is under threat. Antarctica is one of the most rapidly changing environments on the planet due to human activities (36), and soil communities are expected to respond rapidly to these changes (37). Studies conducted in pristine ecosystems relatively free from human disturbance are, therefore, crucial and becoming increasingly difficult to undertake. While previous work demonstrated that microbial community assembly processes rapidly become deterministic on short time scales (14), it is difficult to know whether strong determinism of microbial communities is driven by aboveground selection on microbial communities. Indeed, both plant and bacterial communities change rapidly during succession (38), and plant communities exert strong selection on microbial communities that can lead to a decline in richness and the appearance of environmental filtering in less than 100 years following deglaciation (39, 40). By examining a succession gradient that has been free of aboveground vegetation for millennia, our study suggests that the rapid inclination toward determinism in bacterial community succession is apparently an artifact of aboveground succession. In the absence of vegetation, deterministic processes did increase in importance. but never fully replaced stochastic processes as the strongest driver of microbial community assembly as soils aged. Thus, microbial communities per se might be more strongly dispersal limited than previously assumed.

## MATERIALS AND METHODS

### Sampling methods.

Soil samples were collected from the Shackleton Glacier region from December 2017 to January 2018. A total of 232 soil samples were collected from 12 sites running the length of the glacier. These sites represent a range of elevations (150 to 2221 m asl) across a 120-km north-south distance running from the Ross Ice Shelf to the Polar Plateau (Fig. 1). Between 4 to 26 soil samples were collected along transects located at each of the 12 sites to maximize variation in soil characteristics. Soils (0 to 10 cm depth) were collected in sterile polyethylene bags using clean hand trowels. GPS coordinates, photographs of the soil surface, elevation, and other metadata were recorded at the time of soil sample collection. All soils were transported to the field camp in insulated coolers, where they were frozen at –20°C and remained frozen until processed at the University of Colorado, Boulder, CO, USA.

### Soil geochemistry.

Gravimetric soil moisture was measured by weighing 50 g of subsampled soil pre- and postoven drying at 105°C for 24 h. The water-soluble salt data interpreted in this study were reported by Diaz et al. (41, 42). Briefly, soils were leached at a 1:5 soil:DI water ratio for 24 h. The leachate was filtered through a 0.4 μm Nuclepore membrane filter and analyzed for major anions (F-, Cl-, Br-, SO_4_^2−^) on a Dionex ICS-2100 ion chromatograph, and cations (K^+^, Na^+^, Ca^2+^, Mg^2+^) on a PerkinElmer Optima 8300 Inductively Coupled Plasma-Optical Emission Spectrometer (ICP-OES), and nutrients (NO_3_-, NO_2_-, PO_4_^3−^, SiO_2_, NH_3_) on a Skalar San++ Automated Wet Chemistry analyzer (41–43). We focused on the subset of soil samples from Diaz et al. (41, 42) that had all biogeochemical measurements within instrumentation detection limits. We did not measure soil organic carbon because it is nearly absent from the Transantarctic Mountains (TAM), being below 0.5% and rarely above 2% (44). We also did not measure pH because it is relatively consistent (7.5 to 9.5) throughout the TAM (45, 46), and historical surveys found near-neutral soil pH at even the most isolated, harshest, saltiest site: Robert’s Massif (47). Further, soil pH and soil water content are nearly colinear in the TAM (45), such that including soil pH in analyses would likely not influence results. We also measured Sr^2+^ and PO_4_^−3^, but excluded these two variables from all analyses because they were below detection limit at most sites.

### Microbial DNA sequencing and analyses.

DNA was extracted from each soil sample using the Qiagen Dneasy Powersoil HTP 96 kit (Qiagen, Germantown, MD, USA), following the manufacturer’s recommendations. Extracted DNA was PCR-amplified using a primer pair that targets the hypervariable V4 region of the archaeal and bacterial 16s rRNA gene (515F/806R), with appropriate Illumina adapters and unique barcode sequences. Amplified product was cleaned and normalized to equimolar concentrations using SequalPrep Normalization Plates (Thermo Fisher Scientific, Carlsbad, CA, USA) and sequences on an Illumina MiSeq using the V2 2 × 150 bp paired-end Illumina sequencing kits (Illumina, San Diego, CA, USA). 16s rRNA gene sequences were processed using the DADA2 pipeline (48) and were quality filtered and clustered into amplicon sequence variants (ASVs) with all sequences in a given ASV sharing 100% sequence similarity. Taxonomic information was assigned to ASVs using a Naive Bayesian classifier (49), which takes the set of ASVs generated and compares them to a training set of reference sequences from the 16s rRNA bacterial and archaeal SILVA database (50, 51). Samples that met a threshold of 586 reads per sample were included in downstream analyses. A total of 86 samples met this threshold and had the complete set of corresponding geochemical data, and these 86 samples were used in all downstream analyses.

### Hypothesis 1: The environment and spatial arrangement control microbial community composition.

Our first hypothesis predicts that microbial community composition will be controlled by both the soil environment and the spatial arrangement of sites. We tested this hypothesis by analyzing the relationship between microbial species richness and soil properties, by analyzing the relationship between soil properties, spatial distribution, and microbial community composition, and finally by partitioning variance in microbial community composition between the environment and spatial site arrangement.

### Principal-component analysis of soil geochemistry.

First, we collapsed multivariate metrics of soil characteristics into fewer dimensions using a principal-component analysis (PCA) of soil geochemistry and water content. The variables included salts (F- [ppm], Cl- [ppm], SO_4_^2−^ [ppm], Na^+^ [ppm], Mg^2+^ [ppm], K^+^ [ppm], Ca^2+^ [ppm], SiO_2_ [ppm], NO_3_- [ppm], NH_3_ [ppb]), and gravimetric soil moisture. All variables were log-transformed prior to PCA to improve linearity, and log-transformed variables were then all scaled to N(0, 1) distributions to remove the effects of different measurement scales. PCA proceeded via SVD decomposition of the standardized geochemistry matrix (52).

### Rarefaction.

To analyze how whether the environment controlled ASV richness, we first rarefied ASV richness to a standard number of reads to account for differing sampling depths among sites. Individual-level ASV richness was rarefied using the asymptotic equivalent Σ*_i_^S^*(1 – [1 – *N_i_*/*N*]^N^*), where *S* is the total number of ASVs, *N_i_* is the number of reads for the *i^th^* ASV, *N* is the total number of reads in the sample, and N* is the rarefied number of reads (1 to 3,000) (53). We standardized ASV richness at all sites to 3,000 reads, the smallest number of total reads from the final 86 samples, to standardize ASV richness across all sites to the same, minimum number of reads. Standardizing to a higher number of reads would require extrapolation beyond the data for species-poor sites, and rarefaction curves showed that all sites reached an asymptote, such that 3,000 reads sufficiently captured species richness (Fig. S1). All remaining 86 samples (after removing unreliable samples with fewer than 586 reads) had at least 3,000 reads, and we calculated rarefied richness at the smallest number of reads so that no sample had to extrapolate beyond the observed number of reads.

10.1128/msystems.01254-22.1 FIG S1 Rarefaction curves for each site showed clear asymptotes by 3,000 reads, indicating that our sampling had sufficiently captured ASV richness. Red line shows 3,000 reads. Download FIG S1, PDF file, 0.03 MB. Copyright © 2023 Lemoine et al. 2023 Lemoine et al. https://creativecommons.org/licenses/by/4.0/ This content is distributed under the terms of the Creative Commons Attribution 4.0 International license.

### Redundancy analysis of microbial communities.

We then assessed how both the environment and the spatial arrangement of sites affected microbial community composition conducting RDA of Hellinger-transformed microbial communities that included both environmental and spatial predictors. Environmental predictors were the first two soil principal component axes. Spatial predictors were Moran’s Eigenvector Maps (MEMs). To construct MEMs, we first calculated pairwise distances (km) between all sites in a matrix ***D***. Following Legendre and Legendre (52), we set the threshold value for distances at the maximum distance of a minimum spanning tree for ***D***; any pairwise distance above this threshold (as well as the diagonal elements) were set to four times the threshold amount to generate a truncated matrix D*. We then conducted a principal coordinates analysis (PCoA) on D*. From the PCoA, we kept only the eigenvectors associated with positive eigenvalues. We visually inspected these 12 eigenvectors for interpretable spatial patterns by plotting a map of the sites and coloring the points according to eigenvector score. The first four MEMs resulted in interpretable patterns and were thus used in downstream analyses. The remaining eight MEMs did not describe spatial patterns that we could easily categorize or discern and were excluded from subsequent analyses. The RDA included predictors of an environmental matrix ***E*** containing the first two soil principal components and a spatial matrix ***S*** containing the first four MEMs. The RDA returned si*x* axes that cumulatively explained 45% of the variation in fitted site scores. RDA was conducted at both the phylum and ASV levels (Fig. S3).

Following RDA, we used variance partitioning to describe the amount of variation in soil microbial communities accounted for by spatial and environmental variables. Variance partitioning proceeded by creating three RDA models; spatial only (***S***), environment only (***E***), and spatial + environment (***S+E***) predictors. We calculated the variance explained (R^2^) of each model, and used the following equations to isolate the variance due to each component:

$$\text{Spatial variance }={\text{ R}}^{\text{2}}{}_{\text{S}+\text{E}}–{\text{ R}}^{\text{2}}{}_{\text{E}}$$

$$\text{Environmental variance }={\text{ R}}^{\text{2}}{}_{\text{S}+\text{E}}–{\text{ R}}^{\text{2}}{}_{\text{S}}$$

$$\text{Spatial }+\text{ Environmental Variance }={\text{ R}}^{\text{2}}{}_{\text{S}}+{\text{ R}}^{\text{2}}{}_{\text{E}}-{\text{ R}}^{\text{2}}{}_{\text{S}+\text{E}}$$

### Hypothesis 2: Deterministic processes should be strongest in the harshest soils.

We tested the hypothesis that deterministic assembly processes would be strongest in the harshest soils by partitioning variation in *β*-diversity into “deterministic” and “stochastic” variability for every site along the environmental gradient. First, we partitioned the variation in *β*-diversity using the method of Ning et al. (13) with the following procedure:

1. We calculated neutral regional frequency weights ***w_f_*** by calculating the proportion of sites occupied for each ASV. Proportions were then divided by the proportion sums across all ASVs to normalize into a probability weighting (i.e., Σ***w_f_*** = 1).
2. We calculated neutral regional abundance weights ***w_a_*** by summing the total number of reads for each ASV across all sites, then divided ASV totals by the total number of reads for all ASVs and sites.
3. For each site, we randomly drew species occurrences based on regional frequency weights ***w_f_***. ASV richness was fixed at observed ASV richness so that differences in community composition did not reflect differences in richness.
4. Once null-model species had been chosen for each site, we calculated Dirichlet weighting as α = w_a_*/min(w_a_*), where w_a_* are the regional weights only for those species chosen in Step 3. Relative abundances for the null species were then drawn from a Dirichlet(α) distribution.
5. We then calculated pairwise distances among sites using either Sørensen or Bray-Curtis pairwise site dissimilarities. This procedure resulted in 1,000 estimates of neutral Sørensen and Bray-Curtis pairwise site dissimilarities. We next calculated pairwise dissimilarities based on the observed data ***O***. To partition variation in stochastic (***stoch***), deterministic similarity (***det-a***), and deterministic dissimilarity (***det-b***), we estimated *E*(***d***) as the expected pairwise dissimilarity based on neutral simulations, effectively averaging ***d_ij_*** across all 1,000 simulations. Then,

a. If $O_{\mathrm{ij}}>E{\left(d\right)}_{\mathrm{ij}}$:

$$\det-\mathrm{a=(}O_{\mathrm{ij}}\mathrm{-E}{\left(d\right)}_{\mathrm{ij}}\mathrm{)/}O_{\mathrm{ij}}$$

stoch-a = 1-det-a

b. If $O_{\mathrm{ij}}\;<\;E{\left(d\right)}_{\mathrm{ij}}$:

$$\det-\mathrm{b=(}E{\mathrm{(d)}}_{\mathrm{ij}}-O_{\mathrm{ij}}\mathrm{)/(}1-O_{\mathrm{ij}})$$

stoch-b=1-det-b

c. stoch= 0.5(stoch-a+stoch-b)

These steps yielded one estimate of each component (***stoch***, ***det-a***, ***det-b***) for each site to all other sites. In other words, ***stoch*** was an 86 × 86 matrix that contained the contribution of stochasticity to dissimilarities among all sites. For graphics and analyses, we took the average contribution of ***stoch*, *det-a***, ***det-b*** for every site to all other sites (i.e., averaged by columns) used LOWESS regression to identify the relationships between each component and soil nitrate (i.e., salt) concentrations.

### Hypothesis 3: Environmental filtering should be stronger than dispersal in the harshest soils.

The above null models can only separate out “deterministic” versus “stochastic” assembly processes, and do not reflect the underlying processes. To directly assess how the strength of environmental filtering and dispersal vary along an environmental gradient, we simulated communities following different “rules,” with rules consisting of probability weights corresponding to the process in question. The two processes being tested were dispersal and environmental filtering; thus, we generated probability weights related to dispersal probability and niche suitability.

**(i) Dispersal probabilities.** Dispersal weights (***w_d_***) were taken as the inverse-distance weighted relative abundance for each ASV. That is, the dispersal weight for ASV *k* at site *I* (***w_d-ik_***) was equal to the average relative abundance of ASV *k* at all other sites, weighted by the inverse distance to each site: ***w_d-ik_*** = Σ_j≠1_ (1/***d_ij_***)***x_jk_***/Σ_j≠1_ (1/***d_ij_***), such that distant sites with high abundances were down-weighted, while nearby sites with high abundances were up-weighted, as the most likely correspond to “source” populations.

**(ii) Environmental probabilities.** Environmental weights (***w_e_***) were calculated using a support vector machine learning algorithm (Python module *scikit-learn*). Prior to analyses, relative abundance data were converted to presence/absence data. We then used the environmental matrix consisting of all principal components to predict the probability of occurrence of each ASV at each site. The SVC function from *scitkit-learn* uses a radial basis function for the kernel. The probability predictions are generated using logistic regression against the model scores, and then trained with 5-fold cross validation.

Neutral simulations included four different rules: neutral dispersal wherein probabilities were relative regional frequencies ***w_f_*** described above, neutral abundance wherein probabilities were regional abundances ***w_a_*** described above, dispersal weights ***w_d_***, and environmental weights ***w_e_***. We simulated communities in two steps: first, species were randomly filtered for occurrence according to one of the four rules. Next, for the species that passed the first filter, abundances of those species were randomly generated based on a second rule. The combination of rules reflects seven different assembly processes:

1. Completely neutral (***w_f_*** → ***w_a_***)
2. Environmental filtering for occurrence, neutral determination of abundance (***w_e_*** → ***w_a_***)
3. Dispersal limitation for occurrence, neutral determination of abundance (***w_d_*** → ***w_a_***)
4. Environmental filtering for occurrence, environmental filtering for abundance (***w_e_*** → ***w_e_***)
5. Environmental filtering for occurrence, dispersal limitation of abundance (***w_e_*** → ***w_d_***)
6. Dispersal limitation for occurrence, environmental filtering of abundance (***w_d_*** → ***w_e_***)
7. Dispersal limitation for occurrence, dispersal limitation of abundance (***w_d_*** → ***w_d_***)

For each simulation, we calculated the Sørensen and Bray-Curtis dissimilarities of observed communities to simulation communities. We repeated 1,000 simulations and averaged the dissimilarities for each site across all simulations. The environmental and distance weights were only weakly correlated on the log-scale (Fig. S2).

All analyses were conducted at both the phylum and ASV level. We present phylum-level analyses in text, and ASV-level results are available as supplemental information (Fig. S4 and S5). All code and data used here are available on Figshare (10.6084/m9.figshare.21614226).

10.1128/msystems.01254-22.2 FIG S2 Scatterplots between dispersal and environmental weights on the natural and log-log scales. Download FIG S2, SVG file, 0.1 MB. Copyright © 2023 Lemoine et al. 2023 Lemoine et al. https://creativecommons.org/licenses/by/4.0/ This content is distributed under the terms of the Creative Commons Attribution 4.0 International license.

10.1128/msystems.01254-22.3 FIG S3 Redundancy analyses at the individual ASV level. Download FIG S3, SVG file, 0.8 MB. Copyright © 2023 Lemoine et al. 2023 Lemoine et al. https://creativecommons.org/licenses/by/4.0/ This content is distributed under the terms of the Creative Commons Attribution 4.0 International license.

10.1128/msystems.01254-22.4 FIG S4 Partitioning variance between stochastic and deterministic forces at the ASV level. Download FIG S4, SVG file, 1.2 MB. Copyright © 2023 Lemoine et al. 2023 Lemoine et al. https://creativecommons.org/licenses/by/4.0/ This content is distributed under the terms of the Creative Commons Attribution 4.0 International license.

10.1128/msystems.01254-22.5 FIG S5 Neutral models at the ASV level. Download FIG S5, SVG file, 3.0 MB. Copyright © 2023 Lemoine et al. 2023 Lemoine et al. https://creativecommons.org/licenses/by/4.0/ This content is distributed under the terms of the Creative Commons Attribution 4.0 International license.

### Data availability.

Microbial sequences and soil geochemistry available at the NCBI Sequence Read Archive under BioProject accession number (PRJNA699250).
