# Supplementary material for: Strong Dispersal Limitation of Microbial Communities at Shackleton Glacier, Antarctica
Source: mSystems. 2023 Jan 31;8(1):e01254-22. doi: 10.1128/msystems.01254-22 (PMC9948728; doi:10.1128/msystems.01254-22)
Supplement: TABLE S1 [file msystems.01254-22-s0006.docx]

|  | **Soil PC 1** | **Soil PC 2** |
| --- | --- | --- |
| Elevation | -0.24 | 0.51 |
| F^-^ | -0.28 | 0.14 |
| Cl^-^ | -0.17 | -0.68 |
| SO_4_^2-^ | -0.35 | 0.12 |
| Na^+^ | -0.37 | 0.03 |
| Mg^2+^ | -0.37 | -0.11 |
| K^+^ | -0.36 | 0.04 |
| Ca^2+^ | -0.36 | -0.07 |
| SiO_2_ | -0.09 | -0.08 |
| NO_3_^-^ | -0.34 | -0.17 |
| NH_3_ | -0.21 | 0.22 |
| Soil Moisture | -0.07 | -0.38 |
