# Supplementary figures and images for: Strong Dispersal Limitation of Microbial Communities at Shackleton Glacier, Antarctica
Source: mSystems. 2023 Jan 31;8(1):e01254-22. doi: 10.1128/msystems.01254-22 (PMC9948728; doi:10.1128/msystems.01254-22)

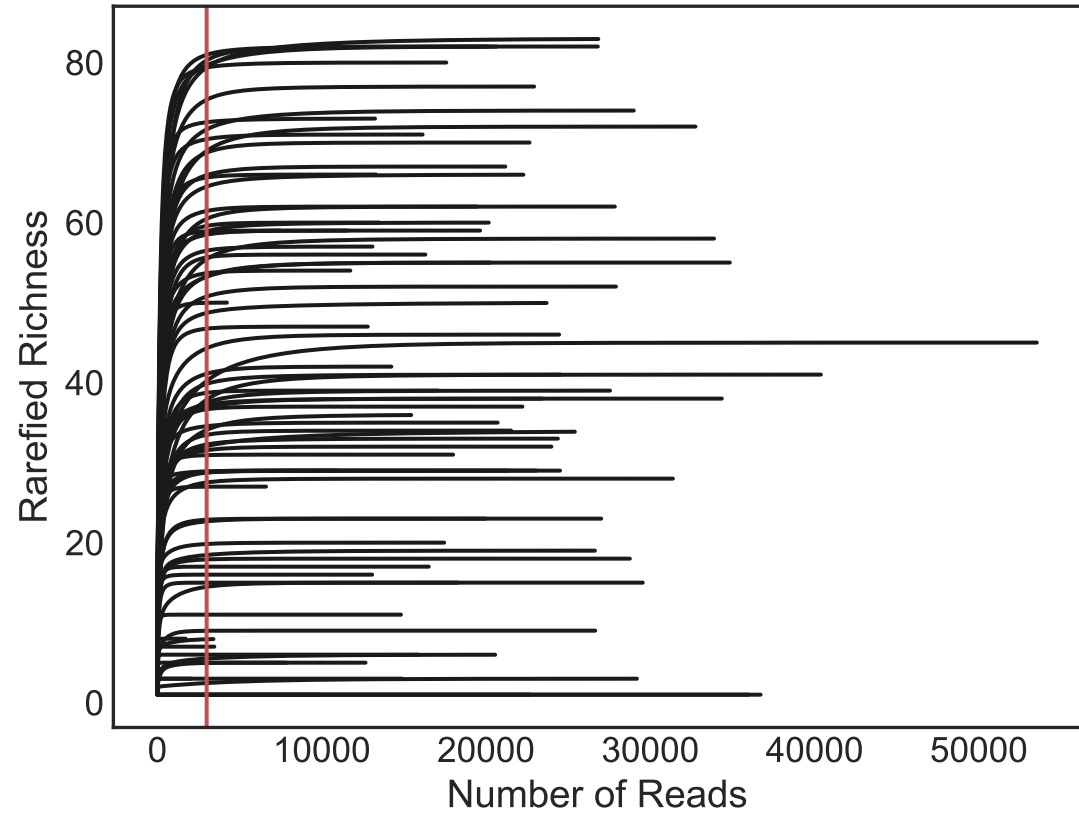

Supplement: FIG S1 [file msystems.01254-22-s0001.pdf]
